# Supplementary material for: The cellular coding of temperature in the mammalian cortex
Source: Nature. 2023 Feb 8;614(7949):725–31. doi: 10.1038/s41586-023-05705-5 (PMC9946826; doi:10.1038/s41586-023-05705-5)
Supplement: Supplementary file 1 — Reporting Summary [file 41586_2023_5705_MOESM1_ESM.pdf]

nature portfolio

Corresponding author(s): James Poulet, Mikkel Vestergaard, Mario Carta

Last updated by author(s): 25/11/22

Reporting Summary

Nature Portfolio wishes to improve the reproducibility of the work that we publish. This form provides structure for consistency and transparency in reporting. For further information on Nature Portfolio policies, see our Editorial Policies and the Editorial Policy Checklist.

Statistics

For all statistical analyses, confirm that the following items are present in the figure legend, table legend, main text, or Methods section.

n/a	Confirmed	
☐	☒	The exact sample size (*n*) for each experimental group/condition, given as a discrete number and unit of measurement
☐	☒	A statement on whether measurements were taken from distinct samples or whether the same sample was measured repeatedly
☐	☒	The statistical test(s) used AND whether they are one- or two-sided<br>*Only common tests should be described solely by name; describe more complex techniques in the Methods section.*
☒	☐	A description of all covariates tested
☐	☒	A description of any assumptions or corrections, such as tests of normality and adjustment for multiple comparisons
☐	☒	A full description of the statistical parameters including central tendency (e.g. means) or other basic estimates (e.g. regression coefficient) AND variation (e.g. standard deviation) or associated estimates of uncertainty (e.g. confidence intervals)
☐	☒	For null hypothesis testing, the test statistic (e.g. *F*, *t*, *r*) with confidence intervals, effect sizes, degrees of freedom and *P* value noted<br>*Give P values as exact values whenever suitable.*
☒	☐	For Bayesian analysis, information on the choice of priors and Markov chain Monte Carlo settings
☒	☐	For hierarchical and complex designs, identification of the appropriate level for tests and full reporting of outcomes
☐	☒	Estimates of effect sizes (e.g. Cohen's *d*, Pearson's *r*), indicating how they were calculated

Our web collection on statistics for biologists contains articles on many of the points above.

Software and code

Policy information about availability of computer code

Data collection	For running behaviour experiments a custom script was created in Labview (16.0f5, National Instruments, USA) or with the Bpod system (1.8.2, Sanworks, USA). A script was created in Python for widefield and two photon imaging together with the use of ThorImageLS software (v4.0.2019.8191). Neuralynx (Cheetah) was used for electrophysiology data.
Data analysis	Motion correction of two photon data, identification of putative neurons and calculation of ΔF/F was done using the Suite2p package (v0.9.3) for Python (Pachitariu et al BioRxiv 2017). The extracellular recordings were spike sorted using Kilosort (version, 2)(Pachitariu et al BioRxiv 2016). A script was created in Python to analyze imaging and behavior data using standard techniques described in the methods.

For manuscripts utilizing custom algorithms or software that are central to the research but not yet described in published literature, software must be made available to editors and reviewers. We strongly encourage code deposition in a community repository (e.g. GitHub). See the Nature Portfolio guidelines for submitting code & software for further information.

Data

Policy information about availability of data

All manuscripts must include a data availability statement. This statement should provide the following information, where applicable:

- Accession codes, unique identifiers, or web links for publicly available datasets
- A description of any restrictions on data availability
- For clinical datasets or third party data, please ensure that the statement adheres to our policy

Data are available from the corresponding author upon reasonable request.

Human research participants

Policy information about studies involving human research participants and Sex and Gender in Research.

Reporting on sex and gender	*Use the terms sex (biological attribute) and gender (shaped by social and cultural circumstances) carefully in order to avoid confusing both terms. Indicate if findings apply to only one sex or gender; describe whether sex and gender were considered in study design whether sex and/or gender was determined based on self-reporting or assigned and methods used. Provide in the source data disaggregated sex and gender data where this information has been collected, and consent has been obtained for sharing of individual-level data; provide overall numbers in this Reporting Summary. Please state if this information has not been collected. Report sex- and gender-based analyses where performed, justify reasons for lack of sex- and gender-based analysis.*
Population characteristics	*Describe the covariate-relevant population characteristics of the human research participants (e.g. age, genotypic information, past and current diagnosis and treatment categories). If you filled out the behavioural & social sciences study design questions and have nothing to add here, write "See above."*
Recruitment	*Describe how participants were recruited. Outline any potential self-selection bias or other biases that may be present and how these are likely to impact results.*
Ethics oversight	*Identify the organization(s) that approved the study protocol.*

Note that full information on the approval of the study protocol must also be provided in the manuscript.

Field-specific reporting

Please select the one below that is the best fit for your research. If you are not sure, read the appropriate sections before making your selection.

☒ Life sciences ☐ Behavioural & social sciences ☐ Ecological, evolutionary & environmental sciences

For a reference copy of the document with all sections, see nature.com/documents/nr-reporting-summary-flat.pdf

Life sciences study design

All studies must disclose on these points even when the disclosure is negative.

Sample size	Sample sizes were not predetermined based on statistical methods but on sizes described in recent literature and experience from previous studies (Milenkovic et al., Nat Neuroscience 2014; Chet et al., Current biology 2021; Chet et al., Science 2011; Ohki et al., Nature, 2005, Peng et al., Nature, 2015).
Data exclusions	Imaging experiments: In a few cases imaging sessions were excluded due to poor optical access, movement or very low number of trials. Behavioral experiments: No data were excluded from animals able to reach a minimal performance on the detection task (>70% correct and <30% false alarm).
Replication	All attempts of replication were successful. Imaging data were replicated in several cohorts of mice. Behavioral data was replicated in two cohorts.
Randomization	Imaging experiments: Allocation to different groups was random. Stimulation temperatures given were in general interleaved. Behavioral experiments: Allocation to different groups was random.
Blinding	Experimenter was not blind to the genotype of the animal. The initial phase the analysis of behavior data was done blind to the genotype.

Reporting for specific materials, systems and methods

We require information from authors about some types of materials, experimental systems and methods used in many studies. Here, indicate whether each material, system or method listed is relevant to your study. If you are not sure if a list item applies to your research, read the appropriate section before selecting a response.

Materials & experimental systems

n/a	Involved in the study	
☐	☒	Antibodies
☒	☐	Eukaryotic cell lines
☒	☐	Palaeontology and archaeology
☐	☒	Animals and other organisms
☒	☐	Clinical data
☒	☐	Dual use research of concern

Methods

n/a	Involved in the study	
☒	☐	ChIP-seq
☒	☐	Flow cytometry
☒	☐	MRI-based neuroimaging

Antibodies

Antibodies used	mouse anti-Gad67 (cat. no.: clone 1G10.2, #MAB5406; Millipore; 1:800)<br>chicken anti-GFP (cat. no.: ab13970; Abcam; 1:250)<br>mouse anti-NeuN (cat. no.: clone A60 #MAB377; Millipore; 1:100)<br>Cy3 goat anti mouse (A-21422; Invitrogen; 1:250)<br>A488 goat anti chicken (A-11039; Invitrogen; 1:250)
Validation	Antibodies were only used if validated by the manufacturer via their website. All antibodies used are standard in the field.<br><br>All antibodies used in this study were validated as described a the following websites (and refrences therein):<br>hhttps://www.merckmillipore.com/FR/fr/product/Anti-GAD67-Antibody-clone-1G10.2,MM_NF-MAB5406;<br>https://www.abcam.com/gfp-antibody-ab13970.html;<br>https://www.merckmillipore.com/FR/fr/product/Anti-NeuN-Antibody-clone-A60,MM_NF-MAB377;<br>https://www.thermofisher.com/antibody/product/Goat-anti-Mouse-IgG-H-L-Cross-Adsorbed-Secondary-Antibody-Polyclonal/A-21422;<br>https://www.thermofisher.com/antibody/product/Goat-anti-Chicken-IgY-H-L-Secondary-Antibody-Polyclonal/A-11039.

Animals and other research organisms

Policy information about studies involving animals; ARRIVE guidelines recommended for reporting animal research, and Sex and Gender in Research

Laboratory animals	In the study male and female mice >2 month old of the the following strains were used: Thy1-GCaMP mice (C57BL/6J-Tg(Thy1-GCaMP6s)GP4.3Dkim/J) and VGAT-CHR2 (B6.Cg-Tg(Slc32a1-COP4*H134R/EYFP)8Gfng/J. They were kept in a 12:12 hour dark:light cycle with experiments performed during the light phase of the cycle. Mice were housed in groups at 22-24°C temperature and 45-55% humidity with ad libitum access to food and water unless stated.
Wild animals	No wild animals were used in the study
Reporting on sex	We used 26 male and 39 female mice and did not select mice for each experiment based on sex. Because the study was not designed to identify sex differences in thermal encoding, our sample sizes do not allow meaningful analysis of sex differences.
Field-collected samples	No field collected samples were used in the study.
Ethics oversight	Approved licenses from Landesamt für Gesundheit und Soziales (LAGeSo), Berlin, Germany

Note that full information on the approval of the study protocol must also be provided in the manuscript.
